# A Thermodynamic Model of Diameter- and Temperature-dependent Semiconductor Nanowire Growth

**DOI:** 10.1038/s41598-017-15077-2

**Published:** 2017-11-08

**Authors:** Xinlei Li, Jun Ni, Ruiqin Zhang

**Affiliations:** 1 Beijing Computational Science Research Center, Beijing, 100193 China; 2 MOE Key Laboratory of Laser Life Science, Institute of Laser Life Science, College of Biophotonics, South China Normal University, Guangzhou, 510631 China; 3 State Key Laboratory of Low-Dimensional Quantum Physics, Department of Physics, Tsinghua University, Beijing, 100084 China; 4 Department of Physics, City University of Hong Kong, Hong Kong SAR, China; 5 Shenzhen Research Institute, City University of Hong Kong, Shenzhen, 518057 China

## Abstract

Creating and manipulating nanowires (NWs) with controllable growth direction and crystal orientation is important to meeting the urgent demands of emerging applications with designed properties. Revealing the underlying mechanisms of the experimentally demonstrated effects of NW diameter and growth temperature on growth direction is crucial for applications. Here, we establish a thermodynamic model to clarify the dependence of NW growth direction on diameter and temperature via the vapor-liquid-solid growth mechanism, enabling analysis of NW critical length between unstable and stable states. At a small critical length, NWs with a large diameter or grown at low temperature tend to grow along the <111> direction, while at a large critical length, NWs with a small diameter or grown at high temperature favor the <110> direction. Specific growth parameters of ZnSe NW have been obtained which can guide the design of functional NWs for applications.

## Introduction

Semiconductor nanowires (NWs) have attracted considerable interest because of their unique physical properties that are not seen in bulk materials^1,2^ and are suitable for applications in a wide range of areas, including biosensors and electronic/optoelectronic devices^3–6^. The properties of NWs depend significantly on their morphologies. In order to improve the physical properties for device application, controlling the size, shape, and growth direction of NWs is a very important and challenging task for both experimentalists and theorists in nanotechnology^7–14^. Investigation of NW growth processes is of fundamental significance to flexible control of the self-assembly and synthesis processes of NWs for potential applications. For instance, Si NWs synthesized via  the vapor-liquid-solid (VLS) technique, are the ideal model systems for studying NW growth and  have been studied intensively in the past decade^7,11^.

The VLS mechanism can be studied by introducing a catalytic liquid alloy droplet, which incorporates vapor atoms by adsorptions. The continuous adsorption causes supersaturation in the alloy droplet, and crystal growth can subsequently occur from nucleated seeds at the liquid-solid interface. In this mechanism, the metal catalyst forms liquid alloy droplets at a high temperature by adsorbing vapor components or intermixing with substrate. Temperature or vapor pressure fluctuation, among other reasons, can lead to the alloy droplet becoming supersaturated; that is, it becomes a solution in which the actual concentration of the components is higher than the equilibrium concentration. This then drives the nucleation and growth of the component at the liquid-solid interface to achieve the minimum free energy of the alloy system. The one-dimensional structure continuously grows if the vapor components are supplied. This growth is known to follow the VLS mechanism^15^, because it involves vapor, liquid, and solid phases. The VLS method has become the most powerful single-crystal NW growth technique due to its high throughput, relatively low cost, and its applicability to various semiconductor materials. The control of NWs’ growth direction and crystal orientation, which determine their electrical, optical, and mechanical properties, is a major issue in the VLS technique^16^. NW diameter and growth temperature have been recognized as the dominant factors determining growth direction. Schmidt *et al*. have demonstrated that epitaxially grown Si NWs with diameters larger than 40 nm tend to the <111> direction, but those with diameters smaller than 20 nm grow along the <110> direction^7^. Similar features are found in ZnSe NWs:^8^ most ZnSe NWs with diameters greater than 20 nm tend to grow along the <111> direction on GaAs (111) or (001) substrates, and ZnSe NWs with diameters of 10–20 nm or even smaller may grow along the <110> or <112> directions. Meanwhile, researchers have found that the growth direction of ZnSe NWs is also determined by the  growth temperature^9^. In particular, for ZnSe NWs with a fixed diameter, a high growth temperature leads to the <112> or <110> growth directions, while a low growth temperature results in growth along the <111> direction.

To explain the diameter- and temperature-dependence of growth direction, some models based on the energy analysis of grown NWs estimating the surface and interface energies of NWs have been proposed^7–9,11,14^. These models calculate the total free energy of NWs with different growth directions, and one can find the most favorable growth direction with the lowest free energy. However, one parameter in these models, called the thickness of interface^7^ or the effective critical thickness of the terminal zone^8,9^, is crucial for the calculation of NW energy. The parameter is usually estimated to approximately fit experimental observations and is considered to be a constant for different NW diameters^7–9^. It is as yet unclear what determines the value of the thickness. In fact, the critical thickness in the calculation of NW energy may be influenced not only by growth temperature, but also by NW diameter, because the initial growth stage is also dependent on diameter^11,14^.

Therefore, we establish a thermodynamic model toquantitatively analyze the values of critical thickness, which here is called the critical length of NWs between unstable and stable states. It is found that the critical length of NWs is influenced strongly by NW diameter and growth temperature. Using critical length, we calculate and compare the total free energy of NWs with different growth directions, and find that NWs with a large diameter or grown at low temperature tend to grow along the <111> direction, and NWs with a small diameter or grown at high temperature favor the <110> direction.

## Theoretical model

**Figure 1:**
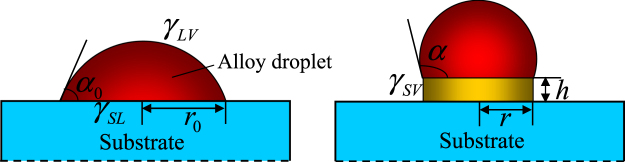
Schematic illustrations of (**a**) alloy droplet formed on the substrate, and (**b**) initial NW growth.

The critical length of NWs is between unstable and stable states. During the initial NW growth process, the solid phase segregation from alloy droplet should obey the layer-by-layer growth mode in the case of homoepitaxial growth based on thermodynamics^17^. However, due to the limits of the alloy droplet, the epitaxial solid phase has a maximal size in the landscape orientation, which is determined by the size of the alloy droplet. Therefore, the growth mode of the solid phase becomes three-dimensional (3D) growth from 2D layer-by-layer growth. The 3D growth mode results in instability of the initial epitaxial solid, which has relatively high free energy. Figure 1 is a schematic diagram of the initial NW growth process. The short initial NW has a height (or length) of *h* and a radius of *r*. The total free energy change caused by NW growth can be given by the equation:

1 $${\rm{\Delta }}G=-{g}_{v}V+{\gamma }_{SV}{S}_{SV}+{\gamma }_{LV}({S}_{LV}-{S}_{0LV})+({\gamma }_{SV}-{\gamma }_{SL})({S}_{0SL}-{S}_{SL})$$

where ${g}_{v}$ is the difference in Gibbs free energy per unit volume expressed by $RT/{V}_{m}{\rm{ln}}(C/{C}^{eq})$ where $R$, $T$, ${V}_{m}$, $C$, and ${C}^{eq}$ are the gas constant, absolute temperature, NW mole volume, the silicon concentration of the solid, and the liquid line of the Au-Si phase diagram, respectively. ${\gamma }_{SV}$ and ${\gamma }_{LV}$ are the surface energy densities of the sides of the NW and the alloy droplet respectively, ${\gamma }_{SL}$ is the liquid-solid interface energy between the alloy droplet and the NW, ${S}_{SV}$ is the surface area of the sides of the NW, ${S}_{0LV}$ and ${S}_{LV}$ are the surface areas of the alloy droplet before and after growing NW respectively, and ${S}_{0SL}$ and ${S}_{SL}$ are the interface areas of the liquid-solid interface before and after growing NW respectively. The first term is the difference of volume energy caused by phase transformation, the second term represents the increase of NW surface energy, the third term is the increase of surface energy of the alloy droplet, and the last term is the energy change caused by the reduction of the liquid-solid interface.

As Eq. (1) divided by the NW volume and multiplied with a negative sign on both sides, the driving force *F* for NW growth can be expressed as:

2 $$F={g}_{v}-{\gamma }_{SV}{S}_{SV}/V-{\gamma }_{LV}({S}_{LV}-{S}_{0LV})/V-({\gamma }_{SV}-{\gamma }_{SL})({S}_{0SL}-{S}_{SL})/V$$

It is only possible for NW growth to occur when $F > 0$, that is, ${g}_{v}$ should satisfy that

3 $${g}_{v} > {\gamma }_{SV}{S}_{SV}/V-{\gamma }_{LV}({S}_{LV}-{S}_{0LV})/V-({\gamma }_{SV}-{\gamma }_{SL})({S}_{0SL}-{S}_{SL})/V$$

For an NW with a circle-shaped section, as shown in Fig. 1, Eq. (3) becomes:

4 $${g}_{v} > \frac{2}{r}{\gamma }_{SV}+(\frac{{r}^{2}}{1+\,cos\,\alpha }-\frac{{{r}_{0}}^{2}}{1+\,cos\,{\alpha }_{0}})\frac{2}{{r}^{2}h}{\gamma }_{LV}+({{r}_{0}}^{2}-{r}^{2})\frac{1}{{r}^{2}h}({\gamma }_{SV}-{\gamma }_{SL})$$

where ${r}_{0}$ and ${\alpha }_{0}$ are the radius and contact angle of the initial alloy droplet before growing NW respectively, $r$ and $\alpha$ represent the radius and contact angle of the alloy droplet after growing NW respectively, and $h$ is the length of the NW. We find that the right-hand term of the equation tends to infinity when $h$ reaches zero. This means that the initial NW growth is unstable when the length of the NW is less than the critical value. The critical length can be expressed as:

5 $${h}^{* }=\frac{2{\gamma }_{LV}[{r}^{2}/(1+\,cos\,\alpha )-{{r}_{0}}^{2}/(1+\,cos\,{\alpha }_{0})]/{r}^{2}+({\gamma }_{SV}-{\gamma }_{SL})({{r}_{0}}^{2}-{r}^{2})/{r}^{2}}{{g}_{v}-2{\gamma }_{SV}/r}$$

Based on the conservation of droplet volumes during NW growth, $r$ and ${r}_{0}$ are related such that ${r}_{0}/r=\sqrt[3]{f(\alpha )/f({\alpha }_{0})}$, where $f(\alpha )$ is a geometric factor expressed as $f(\alpha )={(1-cos\alpha )}^{2}(2+\,cos\,\alpha )/{sin}^{3}\alpha$. Therefore, Eq. (5) can be expressed simply as:

6 $${h}^{* }=\frac{2{\gamma }_{LV}g(\alpha ,{\alpha }_{0})+({\gamma }_{SV}-{\gamma }_{SL})p(\alpha ,{\alpha }_{0})}{{g}_{v}-2{\gamma }_{SV}/r}$$

where $g(\alpha ,{\alpha }_{0})$ and $p(\alpha ,{\alpha }_{0})$ are geometric factors which can be expressed as$g(\alpha ,{\alpha }_{0})=1/(1+\,cos\,\alpha )-$ ${[f({\alpha }_{0})/f(\alpha )]}^{2/3}/(1+\,cos\,{\alpha }_{0})$ and $p(\alpha ,{\alpha }_{0})={[f({\alpha }_{0})/f(\alpha )]}^{2/3}-1$ respectively.

### Diameter- and temperature-dependent NW growth direction

When NW length exceeds the critical value, the NW can grow in a stable state. In other words, an NW with a length less than the critical value is in an unstable state. During the unstable process, the NW seeks an optimal shape, including growth direction and surface geometry, to meet relatively low free energy (NWs tend to grow in a certain direction through minimizing their total free energy, that is, the NW interface and surface energy)^8,9^. For an NW with radius $r$ and length $h$, the total surface and interface energy is given by:

7 $$E=2\pi rh{\gamma }_{SV}+\pi {r}^{2}{\gamma }_{SL}$$

As $E$ divided by NW volume, and surface and interface energy per volume, $\langle E\rangle$ is expressed as:

8 $$\langle E\rangle =\frac{2{\gamma }_{SV}}{r}+\frac{{\gamma }_{SL}}{h}$$

We choose three typical growth directions, <111>, <110>, and <112>, which often appear in Si and ZnSe NWs^7–9^, as examples to investigate the differences in free energy caused by different growth directions.

An <111> -oriented NW with a hexagonal cross-section has six {110}-type facets. Therefore, the surface and interface energy per volume of the <111> -oriented NW is expressed as:

9 $${\langle E\rangle }_{ < 111 > }=\frac{2{\gamma }_{(110)SV}}{r}+\frac{{\gamma }_{SL}}{h}$$

The <110> -oriented NW has four {111}-type facets and two {100}-type facets. To simplify our calculation, we assume that the average surface energy of the polar $(\bar{1}\bar{1}\bar{1})$/$(111)$ pair planes is ${\gamma }_{(111)}$. Then, the surface and interface energy per volume of the <110> -oriented NW can be expressed as^8^:

10 $${\langle E\rangle }_{ < 110 > }=\frac{2[x{\gamma }_{(111)}+(1-x){\gamma }_{(100)}]}{r}+\frac{{\gamma }_{SL}}{h\,cos\,{35.3}^{\circ }}$$

where *x* is a geometrical parameter representing the percentage of {111} facets in the NW circumference, and 35.3° is the inclination angle of the <110> -oriented NW.

Similarly, the surface and interface energy per volume of the <112> -oriented NW can be written as^8^:

11 $${\langle E\rangle }_{ < 112 > }=\frac{2[{x}^{^{\prime} }{\gamma }_{(311)}+(1-{x}^{^{\prime} }){\gamma }_{(111)}]}{r}+\frac{{\gamma }_{SL}}{h\,cos\,{19.5}^{\circ }}$$

Eqs. (9)–(11) relate to the surface-interface energy and radius for given surface-interface energy density and NW length. In Eq. (6), we investigate the critical length of NWs between unstable and stable states. When an NW is shorter than the critical value, change in growth direction can be triggered for meeting a lower energy state. Therefore, we can compare the values of surface-interface energy for different growth directions with a critical length, ${h}^{* }$.

## Results and Discussion

**Figure 2:**
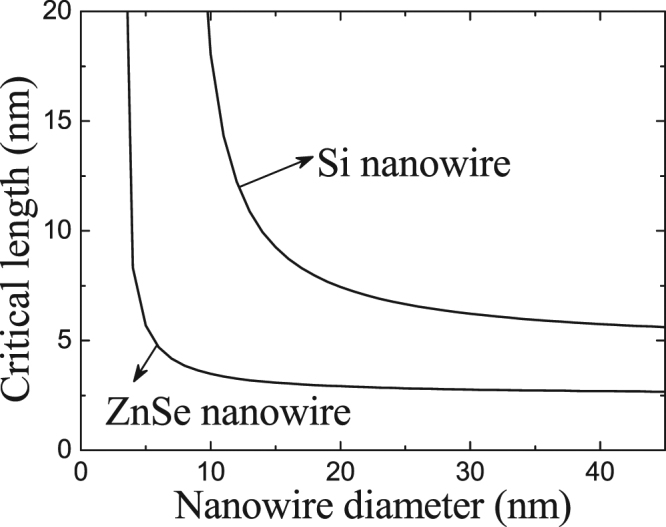
Calculated curve of critical length between unstable and stable states for Si and ZnSe NWs as a function of their diameters at a given growth temperature (400 °C).

Figure 2 shows the values of critical length as a function of the diameter of Si NW and ZnSe NW for a given growth temperature using Eq. (5). One can see that the critical length, ${h}^{* }$, decreases as NW diameter increases. This is because the resistance caused by the surface energy of NWs with a small diameter, that is, $({\gamma }_{SV}{S}_{SV})/V$ in Eq. (2) or $2{\gamma }_{SV}/r$ in Eq. (4), is stronger than that of NWs with a large diameter, so that NWs with a small diameter must be longer than those with a large diameter to achieve a stable state.

**Figure 3:**
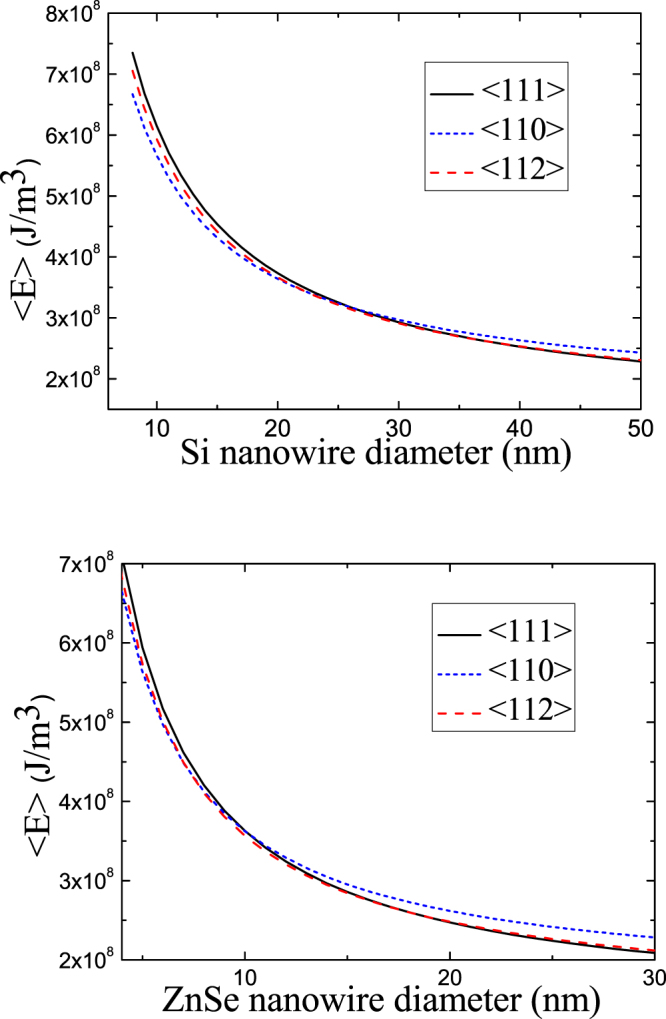
Total free energy per unit volume of <111> -, <110> -, and <112> -oriented NWs as a function of their diameters at a growth temperature of 400 °C for (**a**) Si NWs and (**b**) ZnSe NWs.

Using the values of critical length, total free energy (i.e. the surface and interface energy per unit volume) can be calculated as a function of the diameters for different growth directions of Si and ZnSe NWs, as shown in Fig. 3. One finds that <110> -oriented Si and ZnSe NWs with a small diameter are always in the lowest energy state, and therefore <110> is the preferential growth direction when NW diameter is below a certain value. This is because the side surface of thin NWs is relatively more significant than the interface between droplet and NW. The total free energy of an NW is mainly determined by the surface energy of its sides: <110> -oriented NWs have four {111}-type facets and two {100}-type facets, and their surface energies are lower than those of <110> - and <112> -oriented NWs. Therefore, Si and ZnSe NWs with a small diameter always tend to grow along the <110> direction. However, as NW diameter increases, critical length between unstable and stable states becomes shorter and shorter. When the diameter exceeds a certain value, the interface between droplet and NW becomes more significant than NW side surface due to two reasons: the increase of interface area and the reduction of critical length. In this case, <111> -oriented growth can offer the minimum energy state due to its minimal interface energy. Therefore, <111> is the preferential growth direction for thick NWs, as shown in Fig. 3. Meanwhile, the interface energy and surface energy of <112> -oriented NWs have middling values falling between those of <111> - and <110> -oriented NWs; therefore, <112> -oriented NWs tend to grow when their diameter falls within a certain range of values.

Figure 3(a), regarding the critical diameter of growth direction transition, shows that <110> is the most preferable growth direction for Si NWs with small diameters, under 20 nm. Medium-sized Si NWs (about 20 nm to 35 nm) prefer the <112> growth direction. <111> is the most preferable growth direction for Si NWs with large diameters (over 35 nm). These results agree with the reported experimental observations^7^. Figure 3(b) shows that ZnSe NWs with diameters under 7 nm, 7–16 nm, and larger than 16 nm tend toward <110>, <112>, and <111> growth directions, respectively, in good agreement with the experimental observations and other theoretical models^8^. These agreements strongly suggest that the crossover size of NWs for growth direction transition is governed principally by their surface and interface energies. In particular, NW side surface is relatively more significant than the droplet-NW interface in the case of NWs with a small diameter due to their small interface area and large critical unstable length, so NWs with a small diameter always tend to grow along the <110> direction, which has the lowest surface energy. However, the droplet-NW interface is more significant than NW side surface for NWs with a large diameter, due to their large interface area and small critical unstable length. Therefore, <111> is the preferential growth direction for thick NWs, as it can provide the lowest interface energy.

Comparing these two NWs shown in Fig. 3(a) and (b), we can find that the critical diameter of growth direction transition of ZnSe NWs is significantly smaller than that of Si NWs. The difference is due to that the critical length between unstable and stable states of ZnSe NWs is smaller than that for Si NWs when their diameters are same, as shown in Fig. 2. Small critical unstable length brings about a strong effect of droplet-NW interface, which causes an earlier growth direction transition of ZnSe NWs from <110> direction to <111> direction than that of Si NWs.

**Figure 4:**
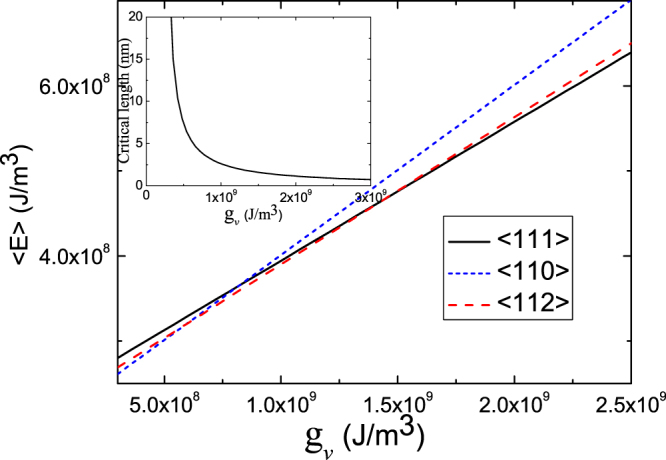
Total free energy per unit volume of <111> -, <110> -, and <112> -oriented ZnSe NWs with a fixed diameter of 10 nm as a function of ${g}_{v}$. The inset shows critical length as a function of ${g}_{v}$.

Beside the influence of NW diameter, growth temperature also plays a critical role in NW growth direction. If we ignore the temperature dependence of surface and interface energy density, the influences of growth temperature are mainly manifested in changes in the energy of volume (i.e. ${g}_{v}$) because of the change of concentration and the degree of super cooling with temperature. According to the thermodynamic principles, low temperature corresponds to strong force from phase transition, and high temperature corresponds to weak force. Therefore, according to Eq. (5), low temperature results in a small critical unstable length and vice versa, as shown by the inset in Fig. (4), which will lead to change in growth direction with the temperature for a fixed NW diameter. With a fixed NW diameter, a large critical unstable length (a high growth temperature) results in <110> growth, but a small critical unstable length (a low growth temperature) may lead to <111> growth. Because the concrete relationships between ${g}_{v}$ and temperature are not clear, we only give an $\langle E\rangle$-${g}_{v}$ plot for different growth directions of NWs with a fixed diameter according to Eqs. (9)–(11). Figure (4) shows the change of total free energy per unit volume as a function of ${g}_{v}$ for ZnSe NWs with a fixed diameter of 10 nm for <111>, <112>, <110> growth directions. One sees that the preferable growth direction with the lowest energy changes from <110> to <112> and then to <111> as ${g}_{v}$ increases (i.e. as temperature decreases). These changes in preferable growth direction are essentially caused by change in critical unstable length, which determines which is more important to total energy, NW side surface or the droplet-NW interface. At high temperatures, because critical unstable length is large, NW side surface is relatively more significant than the droplet-NW interface. So, NWs tend to grow along the <110> direction, which has the lowest surface energy. Contrarily, critical unstable length becomes smaller at low temperatures. The preferable growth direction becomes <112> or <111>, which both have lower interface energy than <110>.

**Figure 5:**
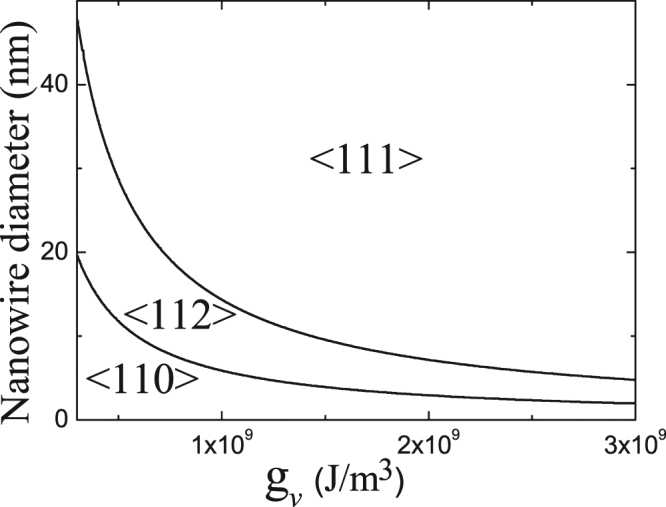
The distribution of ZnSe NW growth directions characterizing the interrelated effects of growth temperature and NW diameter on growth direction.

Therefore, we can show the relationships between growth direction, diameter, and ${g}_{v}$ quantitatively (and temperature qualitatively). Taking ZnSe NWs as examples, Fig. 5 shows the distribution of ZnSe NW growth directions which characterize the interrelated effects of growth temperature and NW diameter on growth direction. We find that when ${g}_{v}$ is fixed (same growth temperature), a large NW diameter results in <111> growth, while a small diameter results in <110> growth. With a fixed NW diameter, a small ${g}_{v}$ (at high temperature) leads to <110> growth, and a large ${g}_{v}$ (at low temperature) results in <111> growth. This agrees well with the reported experimental observations that ZnSe NWs grown at a high temperature (530 °C) favor the <111> direction and those grown at a low temperature (390 °C) favor the <110> direction when their diameters are fixed^9^.

It should be noted that, besides thermodynamic approaches, there are plenty of kinetic methods which can be used for analyzing NW growth processes including the growth rate of the NWs^18–22^. For example, the work by Madras *et al*. represented the first step in understanding the relationship between VLS growth kinetics and NW morphology^19^. They found that single-crystal NWs grown at the fastest rates smoothly, continuously, and randomly vary their growth directions, producing a morphology that is qualitatively different than highly kinked growth. Lungstein *et al*.^20^ demonstrated the controlled growth of SiNWs with different growth orientations, specifically by changing the pressure and found that the growth directions can switch dynamically and reproducibly by changing the growth pressure. Similar results were also reported by Hyuan *et al*.^21^. For nanostructures, thermodynamics and kinetics are two main theories to investigate their growth process. Focusing on NWs, their thermodynamic property is quantified by the energy difference calculated from the free energy during NWs growth, which can reveal the stability of the NWs as well as the final morphology, growth direction, and so on. In contrary, the kinetics is quantified by the rate constant which is associated with the driving energy required for the NWs growth to proceed; that is, the reactivity of the NWs. Although kinetics describes the rates of NW growth and how fast the equilibrium is reached, it gives no information about conditions in which the growth equilibrates. However, thermodynamics can give the information regarding the equilibrium conditions of NWs after the growth taking place. Therefore, thermodynamics has some advantages as compared with kinetics. Compared with the existing thermodynamic models, our work is distinctly new and enriches the knowledge. The critical thickness is the key parameter for the calculation of total energy of NWs in the existing theoretical models including ours. However, in previous reports, the parameter had to be estimated approximately by fitting to experimental data and was considered to be a constant for different NW diameters. Our work solves the problem and provides a way to quantitatively calculate the critical thickness.

Jacobson *et al*. recently found the differences between the growth dynamics of the phases of GaAs NWs, including differences in interface morphology, step flow and catalyst geometry^23^. They explained these differences and the phase selection, using a model that relates the catalyst volume, the contact angle at the trijunction and the nucleation site of each new layer of GaAs. In this way, the V/III ratio controls the droplet volume and crystal structure; in other words, there is a direct correlation between the crystal switch and droplet dimensions (volume, aspect ratio and angle) governed ultimately by the V/III ratio. In our model, we considered the droplet dimensions as constants in the saturation, especially for an elemental state, such as Si NWs. The droplet volume is not governed by the element ratio. But, we are enlightened from the paper to further consider and study the effects of droplet dimensions on the NW growth.

## Conclusions

In summary, we have established a thermodynamic model to address the effects of NW diameter and growth temperature on growth direction using VLS growth mechanism. Using the established model, we have analyzed the critical length of NWs between unstable and stable states. Our results indicate that the critical length of NWs is not a constant, but depends strongly on NW diameter and growth temperature. Large NW diameter and low growth temperature result in a small critical length, and small NW diameter and high growth temperature lead to a large critical length. Using the critical length and considering the contributions of surface energy and interface energy to total NW energy, we find that NWs tend to grow along the <111> direction, which has the lowest interface energy, when they have a large diameter or grow at low temperature, where their critical length is small. However, NWs with a small diameter or grown at high temperature favor the <110> direction, which has the lowest surface energy, where the critical length of NW is large. The good agreement between the established model and experimental observations indicates that the model is quite appropriate for the growth process of an NW synthesized by the VLS growth technique.

## Method

In our calculations, the surface energy density of Si {111}-type facets is about 1.25 J/m^2^ ^24,25^, and the surface energy density of alloy droplets is 0.85 J/m^2^ ^26^. Therefore, with a contact angle of 137° between droplet and Si^27^, an interface energy density of 0.62 J/m^2^ can be obtained based on the Young equation. Furthermore, we use comparison of the relationships of surface energy densities of Si {001}, {113}, and {110} and that of {111} (i.e. the surface energy densities of the Si {001}, {113}, and {110} are normalized with respect to {111} to be 1.11, 1.13, and 1.16, respectively)^28^. Therefore, the surface energy densities of Si {001}, {113}, and {110} are about 1.39 J/m^2^, 1.41 J/m^2^, and 1.45 J/m^2^, respectively. In addition, based on the Au-Si phase diagram and *C*∼1^29,30^, ${\rm{ln}}(C/{C}^{eq})$ is given as about 1.449. For ZnSe NWs, the surface energy density of {111} is about 0.56 J/m^2^, the interface energy density ${\gamma }_{SL}$ is 0.33 J/m^2^, and the surface energy density of the alloy droplet is 0.58 J/m^2^ ^8^.
